# ST-Segment Elevation Myocardial Infarction from Septic Emboli Secondary to Infective Endocarditis by Abiotrophia Defectiva

**DOI:** 10.1155/2020/8811034

**Published:** 2020-07-13

**Authors:** Bashar Khiatah, Sam Jazayeri, John Wilde, Mathew Westfall, Thomas Q. Kong, Amanda Frugoli

**Affiliations:** ^1^Department of Internal Medicine, Community Memorial Hospital, 147 N Brent St, Ventura, CA 93003, USA; ^2^Ventura Cardiology Consultants, 100 North Brent Street, Suite 301, Ventura, CA 93003, USA; ^3^Department of GME Internal Medicine, GME Internal Medicine of Community Memorial Hospital of Ventura, 147 N Brent St, Ventura, CA 93003, USA

## Abstract

This article showcases a young patient who presented with STEMI secondary to septic emboli due to endocarditis with Abiotrophia Defectiva in the setting of a congenital bicuspid aortic valve. We aim to discuss current considerations for STEMI in young individuals including embolism due to IE, especially in patients with known or suspected congenital heart valve disease.

## 1. Introduction

Bicuspid aortic valve “BAV” is the most common congenital cardiac defect and is the most common valvular malformation. Several studies have shown that bicuspid aortic valve (BAV) has a higher incidence of IE than the general population; thus, it is currently considered intermediate-risk cardiac conditions for IE. Infective endocarditis “IE” prevalence has been increasing drastically due to expansion of at-risk persons including a generally aging population, an increase in intravenous drug users (IVDU), increased need for hemodialysis, and an increase in cardiac devices. IE can occur on any single valve or, in rarer cases, may include all valves. It has a wide range of complications that can occur, including prolonged bacteremia, valvular regurgitation, or dehiscence leading to pulmonary edema and heart failure, interruption in the cardiac electrical system, development of metastatic infection focus, and cardioembolic events causing MI or stroke. These are some of the most common findings but others can develop such as mycotic aneurysm, perivalvular abscess, and free wall rupture.

In this case report, we present a unique case of a healthy young male sustaining sudden onset STEMI secondary to infectious endocarditis from Abiotrophia Defectiva in the setting of a congenital bicuspid aortic valve.

## 2. Clinical Case

**Figure 1:**
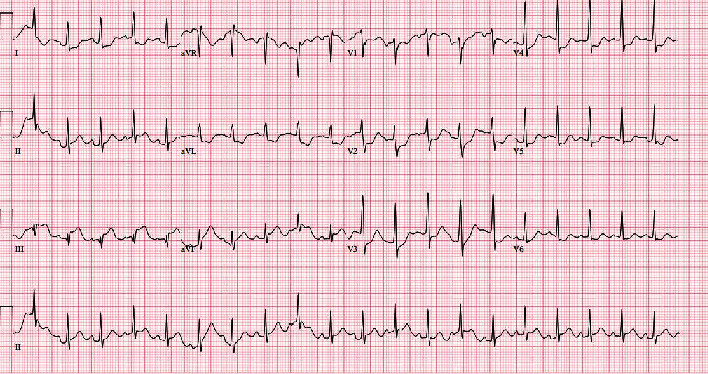
Sinus tachycardia with fusion complexes, ST elevation in lead II, III, AVF, ST depression in V2-V4.

**Figure 2:**
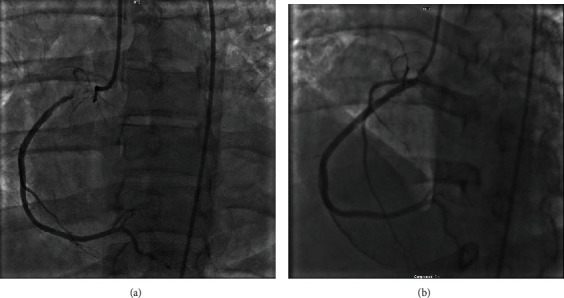
(a) Occlusive septic embolism of the proximal RCA. (b) RCA post stenting.

A 22-year-old healthy male presented to the hospital with complaints of sudden onset substernal chest pain and shortness of breath. Shortly after arrival, ventricular fibrillation occurred and he was defibrillated immediately. ECG (Figure 1) showed ST-segment elevation in the inferior leads, and he was transferred for an urgent angiogram which demonstrated total occlusion of the proximal RCA for which a bare metal stent was placed that successfully restored blood flow (Figures 2(a) and 2(b)).

### 2.1. Differential Diagnosis

STEMI among the young is often divided into the following categories including atheromatous CAD, nonatheromatous CAD (e.g., Kawasaki), sympathomimetic abuse, spontaneous coronary artery or aortic dissection, hypercoagulable states, and cardioembolism.

### 2.2. Investigation

**Figure 3:**
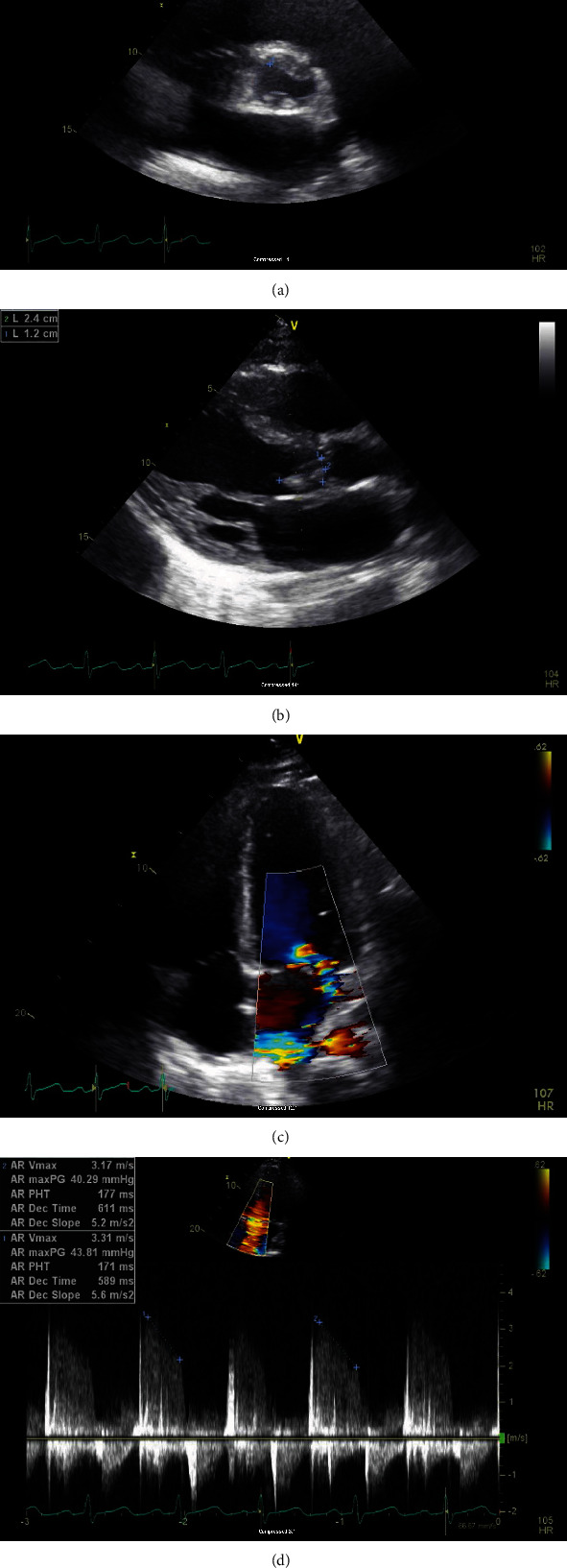
(a) Bicuspid aortic valve. (b) Vegetation on the Aortic valve. (c) Moderate to severe eccentric mitral regurgitation. (d) Severe Aortic valve insufficiency.

**Figure 4:**
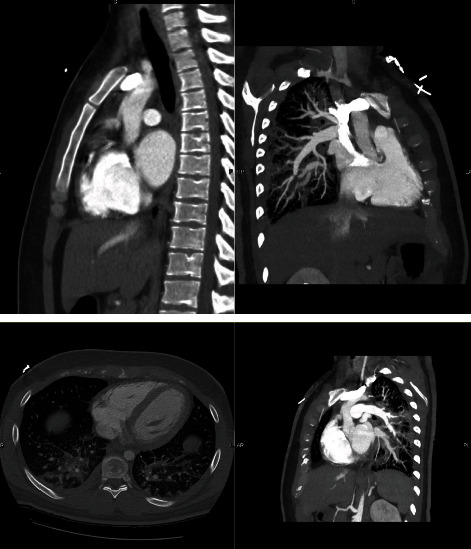
CT Angiography showed perihilar pulmonary infiltrates with ground-glass opacities, mediastinal adenopathy, and splenomegaly.

ECG showed ST-elevation MI in the distribution area of the RCA with elevated troponin I. He was found to have an elevated leukocyte count, creatinine (minor), and BNP levels with mild normocytic anemia. Other basic labs such as TSH, lipid panel, and urine drug screen were within normal limits. Further history investigation after stabilizing the patient revealed weight loss, occasional hemoptysis, and night sweats for the last month for which he was prescribed antibiotics for presumed community-acquired pneumonia. He denied any drug abuse, recent invasive surgery, or recent trauma. His family history was negative for early MI or familial hyperlipidemia. A 2D transthoracic echocardiogram demonstrated left atrial enlargement, a bicuspid aortic valve with a large mobile echodensity measuring 0.9 × 1.7 cm, severe aortic insufficiency, and a malcoaptation of the anterior mitral valve leaflet with moderate to severe eccentric mitral insufficiency and turbulent flow in the left inferior pulmonary vein (Figures 3(a)–3(d)). CTA (Figure 4) was negative for aortic aneurysm or dissection but revealed perihilar pulmonary infiltrates with pulmonary edema, mediastinal adenopathy, and splenomegaly. Blood cultures grew Abiotrophia defectiva from two different bottles. Workup for hypercoagulable disorders was performed and found to be within normal limits. These tests included JAK2, APAS, Factor V Leiden, prothrombin mutation, and DIC panel. Infectious workup for HIV, syphilis, and tuberculosis testing was also negative.

### 2.3. Management

He was treated with empiric antibiotics pending cultures and experienced refractory heart failure due to severe aortic insufficiency and mitral regurgitation. He subsequently underwent successful aortic and mitral valve replacement with bioprosthetic valves and was discharged with an extended course of IV antimicrobials.

## 3. Discussion

ST-segment elevation myocardial infarction in very young patients (≤35 years) is most likely related to illicit or prescribed drug use or to hypercoagulable status such as antiphospholipid, nephrotic syndrome, and factor V Leiden. Smoking, dyslipidemia, and family history remain the most significant traditional risk factors for atheromatous CAD. Spontaneous coronary artery dissection, vasculitis, and septic emboli from an infected valve are also causes of nonatheromatous STEMI. Physicians providing care for such patients are less likely to consider nontraditional cardiac etiologies for myocardial ischemia which may result in delaying diagnosis. The pattern of care and outcomes of the very young with ST-segment elevation myocardial infarction is therefore not well defined [1, 2].

The incidence of IE in the United States is 15 per 100,000 population; however, it is much higher in those with congenital bicuspid aortic heart valves (about one percent of the population) [3]. The incidence of IE among this group is 14 per 10,000 patient-years, which is almost 11 times higher than the general population [4, 5]. Only 0.4% of IE cases are caused by Abiotrophia defectiva, which is a nutritionally variant streptococcus and part of normal oral, urogenital, and intestinal flora [6]. Despite the low incidence rate of IE by Abiotrophia defectiva, it has been reported in the literature in multiple case reports and series demonstrating that the incidence may be uptrending [6]. Notably, 10.5% of all patients with IE by AD had congenital heart disease including bicuspid aortic valve [4]. Among all the patients with IE, only 3% have the complication of acute coronary syndrome [7]. The pathophysiology of ACS in IE could be caused by obstructive coronary embolism, pseudoaneurysm formation, large vegetation blocking the coronary ostia, or compression of the coronary artery by an abscess [7]. It has been reported that a septic embolism of the coronary artery causing a cardioembolic myocardial infarction is a very rare complication of bacterial IE, accounting for <1% of IE complications. Risk factors for embolism in IE include the presence of a mobile vegetation, infection with staphylococci or nonviridans streptococci, vegetation size >10 mm, and previous embolic events [8]. It has been reported that the embolic events were more frequent at early stages [9].

While guidelines for treating infectious endocarditis remain the same [8], the best practices for management of STEMI in IE are not known, and the data is controversial due to the rarity of cases. Although thrombolytic agents have been used successfully in a few cases, the data militate against the use of such agents due to increased risk of severe intracranial hemorrhage, which is thought to be attributed to the high prevalence of silent cerebral infarctions and mycotic aneurysms [10]. Despite limited evidence on the efficacy of PCI, there appears to be a trend in using recanalization as a treatment for STEMI in IE. It has been reported, however, that balloon inflation at the site of the occlusion might cause a displacement of the vegetation and increase the risk of further embolic phenomena and coronary artery mycotic aneurysms [11]. Recently, Nazir S et al. have reported the success rate of each intervention as follows: 56% in balloon angioplasty, 68% in aspiration thrombectomy, and 81% in coronary stenting [10].

Surgery remains indicated in patients who have large vegetations associated with severe valvular disease, those presenting with moderate to severe HF, or in patients with uncontrolled infection. Surgical timing [i.e., urgent versus early (within 48 hours) versus delayed] should be based on individual risk-benefit analysis, with early surgery being strongly indicated when benefits exceed operative risks. Surgery is usually delayed in patients with intracerebral hemorrhage and patients with large cerebral infarction as surgery may pose a significant risk of neurological deterioration and preoperative cerebral bleeding [12].

The choice of replacement with mechanical or tissue prosthesis valves in IE of a native aortic valve remains based on the patient's factors, especially given similar survival and endocarditis recurrence rate in both bioprosthetic and mechanical valves [13]. It has been reported that there is a national trend in using bioprosthetic rather than mechanical valves for aortic valve replacement in IE patients [14]. This is juxtaposed to IE of a native mitral valve, when repair is not an option, replacement with a prosthetic valve is the only choice as there are no established alternatives yet. [13]

In our case, the patient presented with a STEMI requiring urgent angiography and PCI due to occlusive septic embolism of the proximal RCA. Early mitral and aortic valve replacement were indicated due to the large size of the vegetation, severe mitral regurgitation, severe aortic insufficiency, and refractory heart failure in an otherwise young, healthy male with a congenital bicuspid aortic valve.

## 4. Follow-Up

One month postdischarge and after completing a course of six weeks antibiotics regimen, the patient was recovering well.

## 5. Conclusion

Increasing the awareness of the differential diagnosis for STEMI in young patients, especially given the increased incidence of septic coronary artery emboli from infective endocarditis, and determining the most appropriate treatment (thrombolysis vs PCI vs surgery), which itself remains a subject of debate, may help to clarify the standard of care in such patients. Furthermore, given the disparity of evidence and practice, further studies to substantiate the use of PCI in STEMI patients during IE is warranted. Lastly, the consideration of normal flora as a cause of infective endocarditis in a healthy young patient should be considered and further evaluation for undiagnosed congenital heart defect should be pursued.

## Abbreviations

STEMI: ST-elevation myocardial infarction
ROSC: Return of spontaneous circulation
CAD: Coronary artery disease
ECG: Electrocardiogram
RCA: Right coronary artery
DIC: Diffuse intravascular coagulation
CTA: Computed tomography angiography
ACS: Acute coronary syndrome
PCI: Percutaneous coronary intervention
IE: Infective endocarditis.

## Additional Points

*Learning Objectives.* (i) Review the differential diagnosis for STEMI in very young patients. (ii) Treatment options for STEMI secondary to IE.
